# Editorial: Vascular Endothelial Glycocalyx in Cardiovascular Disease

**DOI:** 10.3389/fcvm.2022.952022

**Published:** 2022-07-01

**Authors:** Hiroyuki Tomita, Kodai Suzuki, Masanobu Komatsu, Hideshi Okada

**Affiliations:** ^1^Department of Tumor Pathology, Gifu University Graduate School of Medicine, Gifu, Japan; ^2^Department of Surgery, Columbia University, New York, NY, United States; ^3^Department of Cancer and Blood Disorders Institute, Johns Hopkins All Children's Hospital, St. Petersburg, FL, United States; ^4^Department of Surgery, Johns Hopkins All Children's Hospital, St. Petersburg, FL, United States; ^5^Department of Emergency and Disaster Medicine, Gifu University Graduate School of Medicine, Gifu, Japan

**Keywords:** endothelium, endothelial glycocalyx, cardiovascular disease, vascular biology, microcirculation

The endothelial glycocalyx coats the surface of all healthy endothelial structures and plays a key role in microvascular and endothelial physiology. This structure helps regulate the microvascular tone and endothelial permeability, maintain an oncotic gradient across the endothelial barrier, regulate leukocyte adhesion/migration, and inhibit intravascular thrombosis (1, 2).

The endothelial glycocalyx may get injured due to several reasons. In the clinical syndromes of systemic inflammation, including sepsis, ischemia/reperfusion, and prolonged hyperglycemia, diffuse and persistent changes in the glycocalyx are associated with widespread endothelial dysfunction, altered permeability, as well as impaired oxygen and nutrient delivery to cells. Several previous reports have suggested the association of endothelial glycocalyx injury with severe diseases such as acute kidney injury, chronic kidney disease, and cardiovascular disease (3–5). In addition, chronic conditions such as diabetes, aging, and hypertriglyceridemia injure the endothelial glycocalyx structure and cause degradation. The ability to directly treat and protect the endothelial glycocalyx would suggest an extremely important finding in this context.

Further basic and clinical research is important to advance our knowledge of endothelial glycocalyx and cardiovascular disease. In this Research Topic, four original research papers (Hatanaka et al.; Mitsuda et al.; Shinohara et al.; Chevalier et al.) and two reviews (Haymet et al.; Milusev et al.) have been described as follows.

In sepsis, endothelial cell damage may be a common basis for multiorgan failure. In the septic state, the accumulation of proteases promotes the shedding of proteoglycans such as syndecan-1(SDC-1) from the endothelial surface, leading to leukocyte adhesion to the vessel wall increased vascular permeability, and intravascular coagulation (6). Hatanaka et al. reported a study where they enrolled 100 consecutive patients with suspected infectious diseases admitted to the intensive care unit of their university hospital. Their serum SDC-1 concentrations were measured using an in-hospital enzyme-linked immunosorbent assay; differences in serum SDC-1 concentrations between survivors and non-survivors on day 28 were analyzed. Correlations between serum SDC-1 and coagulation markers were also analyzed. Serum SDC-1 levels in non-survivors were significantly higher than in survivors on days 1 and 3. Among multiple organ failure, renal failure was significantly correlated with serum SDC-1. Recovery was delayed compared to patients with serum SDC-1 <21.4 ng/ml. Persistent thrombocytopenia and fatal outcomes were associated with patients with suspected sepsis.

Alterations in the glycan structure of the endothelial glycocalyx cause endothelial dysfunction and contribute to the exacerbation of peripheral vascular disease. Therefore, exploring their ultrastructure is a priority to assess the extent of injury under physiological conditions and evaluate the impact of therapeutic approaches. Chevalier et al. performed intravenous perfusion of rats with a lysate containing an aldehyde oxidant enriched with lanthanum ions. This improves the staining of the glycocalyx and allows the detection of elastic and inelastic scattered electrons to delineate the glycocalyx by transmission electron microscopy (TEM). Scanning electron microscopy (SEM) has been applied to resin-embedded serial sections, enabling rapid and efficient large-area imaging and correlative TEM observation of the ultrastructure. In addition, they performed 3D tomography with a dual-beam focused-ion-beam SEM (FIB-SEM) to show the dynamic features of the glycocalyx. Overall, their study provides an effective means of combining SEM, TEM, and FIB-SEM tomography approaches on the same sample.

Currently, very little information is available on the recovery of glycocalyx after sepsis. Shinohara et al. developed a model that can reproduce glycocalyx recovery in mice using *in vivo* imaging and a dorsal skinfold chamber (DSC). Direct visualization of endothelial glycocalyx thickness and leukocyte-endothelial interactions showed that degraded glycocalyx recovered within 24 h. In contrast, indirect indicators of recovery from sepsis, such as body weight and blood pressure, required longer recovery times. Consequently, this mouse model may help study the refractory angiopathy after sepsis.

Mitsuda et al. performed animal experiments on the “obesity paradox,” which means that a higher BMI appears to increase the survival of patients with sepsis (7–9). They hypothesized that endothelial glycocalyx in obese mice is thicker and more resistant to inflammatory stress than that in non-obese mice. Thus, they used intravital microscopy to explore the differences in vascular endothelial glycocalyx in three groups of mice fed diets with different fat concentrations. The results showed that the mean index of the glycocalyx in the endothelium, defined as the glycocalyx index, was significantly higher in mice on the high-fat and medium-fat diets than in those on the low-fat diet. The data shows that the intrinsic glycocalyx index would play an important role in the obesity paradox.

Thus far, glycocalyx has been studied in static and perfused *in vitro* models, and imaging data, evaluation of barrier function, and interaction of blood components with the endothelial monolayer have been reported. The model incorporating all of these features simultaneously would undoubtedly enhance the study of microvascular lesions. Haymet et al. summarized a series of *in vitro* models described in the previous studies that target the glycocalyx layer, their limitations, and the potential for further development in this area.

Milusev et al. have focused on the physiological functions of the glycocalyx in their review. Particularly, it discusses how the loss of endothelial glycocalyx integrity is associated with cardiovascular risk factors such as hypertension, aging, diabetes, and obesity, and contributes to the development of thrombotic inflammatory conditions. They discussed the role of glycocalyx components in the regulation of inflammatory responses in addition to potential therapeutic interventions aimed at maintaining or restoring endothelial glycocalyx.

Overall, various original papers and reviews discussed here offer valuable insights and future perspectives on endothelial glycocalyx and cardiovascular diseases.

## Author Contributions

All authors listed have made a substantial, direct, and intellectual contribution to the work and approved it for publication.

## Conflict of Interest

The authors declare that the research was conducted in the absence of any commercial or financial relationships that could be construed as a potential conflict of interest.

## Publisher's Note

All claims expressed in this article are solely those of the authors and do not necessarily represent those of their affiliated organizations, or those of the publisher, the editors and the reviewers. Any product that may be evaluated in this article, or claim that may be made by its manufacturer, is not guaranteed or endorsed by the publisher.
